# The Future of Moringa Foods: A Food Chemistry Perspective

**DOI:** 10.3389/fnut.2021.751076

**Published:** 2021-11-02

**Authors:** Silke Grosshagauer, Philip Pirkwieser, Klaus Kraemer, Veronika Somoza

**Affiliations:** ^1^Department of Physiological Chemistry, Faculty of Chemistry, University of Vienna, Vienna, Austria; ^2^Leibniz Institute for Food Systems Biology at the Technical University of Munich, Freising, Germany; ^3^Sight and Life Foundation, Basel, Switzerland; ^4^Department of International Health, Johns Hopkins Bloomberg School of Public Health, Baltimore, MD, United States; ^5^Chair of Nutritional Systems Biology, School of Life Sciences Weihenstephan, Technical University of Munich, Freising, Germany

**Keywords:** *Moringa oleifera*, food supplements, food safety, safety assessment, nutrient profile, anti-nutrients, vitamin source

## Abstract

The tree *Moringa oleifera Lam*. provides its leaves, pods, flowers and seeds for human nutrition. The chemical profile of all these Moringa products varies substantially, not only among the different parts of the plants used. Cultivating, processing as well as storage conditions chiefly determine the contents of nutrients and anti-nutritive constituents. Anti-nutrients, e.g., phytic acid or tannins, are present in notable amounts and may affect micronutrient bioavailability. Although *Moringa oleifera* products have been promoted for several health benefits and are discussed as an alternative treatment in various diseases, risk assessment studies evaluating contamination levels are scarce. Recent investigations have demonstrated alarming contents of heavy metals, polycyclic aromatic hydrocarbons and mycotoxins in *Moringa oleifera* products, indicating the need for a comprehensive risk assessment and contingent legal regulation of these products. In this mini review, we briefly outline pivotal, food chemistry and nutrition related data on Moringa preparations in order to stimulate in-depth research to close the presented knowledge gaps.

## Introduction

The tree *Moringa oleifera Lam*. belongs to the family Moringaceae, genus Moringa, also known as “horse radish tree,” “drumstick tree” or simply as “moringa.” *Moringa oleifera* is native to India but is also prevalent in tropical and subtropical regions of Africa, Asia, Central and South America. Its multipurpose use as food and feed, dietary supplement or even as functional ingredient of cosmetic products has led to an increased cultivation and global trade (1).

In 2018, the market of Moringa products was evaluated with USD 5.5 billion globally, which is considerably lower than the market size for algae products of USD 32.6 billion in 2017. Nevertheless, the global market of Moringa is expected to rise in upcoming years. Products include leaf powder, which accounted for the largest market share of Moringa products with 30%, but also leaf tea, oil and seeds (1).

Due to its high protein content, its richness in vitamins and minerals and the low demanding cultivation conditions, *Moringa oleifera* is often promoted as a promising plant to combat malnutrition (2). The products of the so-called “miracle tree” are also discussed for medicinal usage, therefore it clearly needs a more critical observation of undesired side-effects, but also a closer look at the bioavailability of nutrients (3). As seen for other complementary foods, increasing supplementation with, e.g., Moringa leaf powder, negatively correlated with the sensory quality and therefore consumer acceptance (4). Ultimately, the herein discussed differences in nutritional qualities may be associated with consumer acceptance in the future. In this regard, variations found in the different plant parts [e.g., described for polyphenol composition (5)], in wild type vs. domesticated plants, or after different preparation processes may significantly affect both health promoting properties and sensory quality. Chodur et al. (6), for instance, reported that domesticated Moringa plants could be distinguished clearly from a wild type not only through a higher antioxidant potential, but also by a milder, non-bitter taste due to different glucosinolate compositions. Hence, pivotal food quality and safety related data on Moringa preparations are outlined briefly in this mini review in order to stimulate in-depth research for closing the presented knowledge gaps.

### Reported Health Effects for Type 2 Diabetes

A supportive effect in type 2 diabetes treatment represents one of the many health benefits for which *Moringa oleifera* products are promoted and will be discussed in this chapter. Other health-promoting properties, such as anti-inflammatory or antioxidant effects and immune regulatory bioactivities, have been thoroughly covered elsewhere [e.g., by Lin et al. (7), Xiao et al. (8), and Afzal et al. (9)] and would go beyond the scope of this perspective.

*Moringa oleifera* leaves showed glucose lowering effects in various animal studies, which were suggesting this herbal product for the treatment of type 2 diabetes either instead of metformin, a commonly used drug for type 2 diabetes treatment, or in combination with anti-diabetic drugs (10). In one of these studies showing most pronounced effects, six groups of alloxan-induced diabetic Wistar rats plus one group of normoglycaemic Wistar rats (group I) were treated either with distilled water (group I, II), different amounts of an ethanolic *Moringa oleifera* leaf extract without (group III, IV) or with the addition of 150 mg/kg metformin (group V, VI), or with metformin alone (group VII). After 4 weeks, a significantly lower fasting blood sugar level (FBS) of 307 ± 31 mg/kg could be observed in rats administered with 400 mg/kg *Moringa oleifera* leaf extract (group III) compared to the mean FBS of 477 ± 17 mg/kg in the diabetic control group (*p* < 0.05). The glucose levels could even be more reduced to 252 ± 41 mg/kg when receiving 800 mg/kg *Moringa oleifera* leaf extract (group IV) over 4 weeks. The greatest lowering effect of FBS levels could be obtained in group V and VI, when the diabetic rats were either treated with 400 mg/kg or 800 mg/kg *Moringa oleifera* leaf extract, respectively, plus the addition of 150 mg/kg metformin. After 7 days, the FBS level reached 253.0 ± 35.13 mg/kg and 168.5 ± 21.19 mg/kg in group V and VI, respectively, and continued to decrease to 100.6 ± 15.14 mg/kg and 80.8 ± 5.43 mg/kg, respectively, after 28 days of treatment (10). Despite these positive outcomes, data is lacking whether consumption of *Moringa oleifera* products might be associated with unfavorable consequences in treated rats. Omabe et al. (11) not only investigated the positive effect of *Moringa oleifera* leaves on blood glucose level, but also revealed a development of metabolic and anion gap acidosis in Alloxan-induced type 2 diabetic rats: Compared to the control group, in which rats were administered with 1.5 mL PBS twice a day over a period of 5 days, a more than 2-fold increase in anion gap could be observed in rats treated twice daily with 200 mg/kg of an ethanolic extract of *Moringa oleifera* leaves soluted in 1.5 mL PBS, and even a 3-fold increase of anion gap was determined when 10 mg/kg of metformin plus 1.5 mL PBS were administered over the same treatment period. Therefore, this alternative treatment might not be recommended for type II diabetic patients who are susceptible to develop acidosis.

As reviewed by Stohs and Hartman (12), a smaller number of human studies on the effects of *Moringa oleifera* exist compared to a constantly growing number of animal studies, which should be interpreted with caution. As an example for human intervention studies, Kushwaha et al. (13) showed a significant decrease of fasting blood glucose levels in postmenopausal women from 107 ± 7 to 92 ± 3 mg/dL after consuming 7 g of *Moringa oleifera* leaf powder over a period of 3 months. Nonetheless, a comprehensive literature search conducted by Owens III et al. (14) emphasizes that in order to build upon these first promising results on the prevention or treatment of Type 2 diabetes in humans, more studies with standardized approaches, e.g., standardized Moringa preparations as well as treatment durations and doses, are required.

### Macronutrient Profiling

In general, the nutrient content of Moringa products chiefly depends on the cultivation conditions, e.g., climate characteristics and soil composition, processing and storage conditions (Table 1). For total protein, mean highest amounts of 22.4% were quantitated in dried Moringa leaves, whereas flowers and immature pods contained slightly lower mean contents of 18.9 and 19.3% of total dry mass, as analyzed by means of the commonly used Kjeldahl method (15). Protein digestibility, as one of the main determinants of the nutritional quality of a dietary protein, has not been investigated *in vivo* so far. However, the non-enzymatic hydrolyzability of ground *Moringa oleifera* leaves was studied *in vitro* by Borges Teixeira et al. (16). The authors analyzed a mean total protein content of 277 mg/g in defatted *Moringa oleifera* leaf flour by the Kjeldahl method, and quantitated the amount of protein soluble in sodium dodecyl sulfate (40%) and 2-mercaptoethanole (30%), revealing about 60–70% of the total protein not being hydrolyzed under these conditions.

**Table 1:** Macronutrient profile of *Moringa oleifera* leaves, flowers, immature pods and seeds^a^.

**Sample**	**Country**	**Treatment**	**Protein (%)**	**Total fat (%)**	**Fatty acids** **(% of total FA)**	**Dietary fiber (%)**	**Carbo-hydrates (%)**	**References**
Leaves	Mexico	Oven-dried 60 °C, 8 h	22.4	5.0	SFA 29.9 MUFA 7.2 PUFA 63.2 LA 6.1 ALA 56.9	31.0	27.1	(15)
Flowers	Mexico	Oven-dried 60°C, 8 h	18.9	2.9	SFA 31.8 MUFA 26.3 PUFA 42.2 LA 19.0 ALA 23.0	32.5	36.0	(15)
Immature pods	Mexico	Oven-dried 60°C, 8 h	19.3	1.3	SFA 31.6 MUFA 18.4 PUFA 49.9 LA 23.5 ALA 26.2	46.8	25.0	(15)
Leaves (flour)	Brazil	Oven-dried 35°C, 24 h	28.7	7.1	–	–	44.4	(16)
Seeds	Nigeria	Raw	26.7	30.6	–	1.4	32.0	(17)
Seeds	Nigeria	Boiled			–			(17)
		10 min	21.6	10.4		1.9	49.9	
		20 min	28.9	11.3		2.1	36.5	
		30 min	32.0	12.3		2.3	31.8	
Seeds	Nigeria	Roasted			–			(17)
		10 min	25.9	20.5		3.7	41.0	
		20 min	29.0	10.6		3.7	47.6	
		30 min	26.6	20.5		3.8	42.2	

a *Values are presented as means*.

Another quality indicator of dietary proteins is their content of essential amino acids, for which a wider variation was analyzed among Moringa flowers (44% of total protein) and leaves (31% of total protein), with methionine being the limiting amino acid in all Moringa products (15) (Table 2).

**Table 2:** Amino acid pattern of *Moringa oleifera* leaves, immature pods and flowers in mg/g dry weight [adapted from Sánchez-Machado et al. (15)].

**Amino Acid**	**Leaves**	**Immature pods**	**Flowers**
Histidine	7.0 ± 0.4	2.0 ± 0.3	3.1 ± 0.4
Threonine	7.9 ± 0.4	3.3 ± 0.5	5.4 ± 0.2
Tyrosine	4.8 ± 0.9	0.4 ± 0.1	0.4 ± 0.1
Valine	11.3 ± 1.1	4.3 ± 1.0	6.4 ± 0.6
Methionine	1.4 ± 0.3	0.9 ± 0.2	1.0 ± 0.2
Isoleucine	8.9 ± 0.7	3.1 ± 0.4	5.2 ± 0.5
Leucine	17.5 ± 0.2	5.6 ± 0.5	8.7 ± 0.9
Phenylalanine	8.9 ± 0.3	2.3 ± 0.4	3.8 ± 0.5
Lysine	15.3 ± 0.6	2.5 ± 0.6	4.6 ± 0.5
Aspartate	15.8 ± 1.5	7.4 ± 0.3	12.3 ± 0.9
Glutamate	17.1 ± 1.4	14.6 ± 2.3	17.0 ± 2.2
Serine	9.4 ± 0.5	7.5 ± 1.8	7.5 ± 0.4
Glycine	10.3 ± 0.7	4.3 ± 0.5	6.5 ± 0.3
Alanine	12.5 ± 0.6	4.2 ± 0.7	8.1 ± 0.5
Proline	12.4 ± 0.9	4.0 ± 0.6	6.6 ± 0.5
Arginine	12.2 ± 0.8	8.1 ± 2.5	20.1 ± 1.2

Total lipid contents of 5–7% (15, 16), 3% (15) and 1.3% (15) of dry weight have been reported for leaves, flowers and immature pods, respectively, indicating major variations among the different plant parts. With regard to the fatty acid profile, the highest contents of 6% polyunsaturated fatty acids (PUFAs) of total lipids were analyzed in dried leaves, compared to 50 and 42% in immature pods and flowers, respectively. In addition, 7% of the lipid content in Moringa leaves were assigned to monounsaturated fatty acids (MUFAs), whereas saturated fatty acids (SFA) amounted to 30% (15). Although raw *Moringa oleifera* seeds contained more fat than other edible parts of the indigenous tree, with nearly reaching an amount of 31% (17), the lipid content decreased to 20.5% when the seeds were roasted for 30 min and an even higher loss to 12.3% of total fat content was noted when seeds were boiled for 30 min (Table 1). Concerning dietary fiber, Sánchez-Machado et al. (15) reported the highest values for oven-dried immature pods (46.8%), which compares to the amounts found in high dietary fiber sources such as edible seaweed. Leaves and flowers yielded lower amounts, with 31.0 and 32.5%, respectively. Seeds ranged from 3.8 (roasted for 30 min) to 1.4% (raw) (17) (Table 1).

### Micronutrient Profiling

Moringa products are often promoted as nutritional supplements to treat and prevent micronutrient malnutrition such as vitamin A deficiency, which is prevalent in many parts of Africa or South Asia (18). As such tropical and subtropical countries are home to the drumstick tree, several studies investigated the potential of *Moringa oleifera* products as a vitamin A source (16, 17, 19, 20). Borges Teixeira et al. (16) revealed a mean beta-carotene content of 161 μg/g and a mean lutein content of 47 μg/g lyophilized leaf flour. Converting the amount of beta-carotene to retinol equivalents would be equivalent to 26.8 μg RAE per gram leaves. Similar vitamin A levels were obtained in *Moringa oleifera* seeds, with 20 μg/g in the raw samples, 21–28 μg/g in boiled seeds and 17–19 μg/g in roasted samples (17). These results have been summarized in Table 3. Nevertheless, more profound investigations of *Moringa oleifera* leaves are needed to determine how much of the provitamin carotenoids can be converted to retinol. Furthermore, the study of Sriwichai et al. (22), where bioaccessibility was determined by assessing the amount of compounds transferred into an aqueous micellar phase after an *in vitro* digestion procedure, showed that full bioaccessibility of these bioactive compounds cannot be assumed. The beta-carotene content in raw *Moringa oleifera* leaves was 630 μg/g dry matter, but <5 μg/g dry matter were bioaccessible. Different drying methods as well as grinding and encapsulation were applied in order to allow a destruction of the cell wall and show an efficient way to deliver carotenoids or other bioactive compounds. The best results (<2 mg/100 g dry matter) were obtained when moderate drying temperatures (60°C) were combined with subsequent fine grinding. Encapsulation of the dried and grinded powder lead to similar results of bioaccessible beta-carotene. Although a significant improvement of the bioaccessibility of beta carotene could be obtained with these methods, a great part could not be made bioaccessible (22). Considering these findings, claims and statements of a high carotenoid content should be relativized and further studies about bioavailability and bioconversion to vitamin A are indispensable.

**Table 3:** Vitamin contents in differently prepared edible parts of *Moringa oleifera* in μg/g (ready to eat)^1^.

**Sample**	**Treatment**	**Vit. A^**2**^**	**β-carotene^**2**^**	**α-tocopherol**	**Ascorbic acid**	**References**
Leaves (flour)	Oven-dried 35 °C, 24 h		161			(16)
Seeds (flour)	Raw	20.4				(17)
	Boiled	20.9–28.0				
	Roasted	17.3–18.7				
Leaves (powder)	Fresh		183	369	2,710	(21)
	Lyophilized		821^a^	1602^a^	13,256^a^	
	Cabinet-tray dried		823^a^	1652^b^	5,908^c^	
	Micro-oven dried		659^c^	1355^c^	8,729^b^	
	Oven-dried		778^d^	1596^a^	5,450^d^	
	Sun-dried		544^d^	1123^d^	5,100^d^	

1 *Values are presented as means or range; different superscript letters (each column) represent significant differences (p < 0.05) between dehydration methods*.

2 *1μg retinol activity equivalent (RAE) equals 1 μg preformed retinol, or 2 μg β-carotene supplement in oil, or 12 μg β-carotene, or 24 μg other pro-vitamin A carotenoids*.

Vitamin A could help to release iron from iron stores and therefore help to reduce anemia. Indeed, in a study by Zimmermann et al. (23) vitamin A supplementation to vitamin A deficient children supported hematopoiesis. Instead of direct vitamin A supplementation, Boateng et al. (19) analyzed the hemoglobin stores and some growth indicators in infants aged between 8 and 12 months in the Eastern region of Ghana after receiving complementary foods fortified with *Moringa oleifera* leaf powder over 4 months. The 237 participating infants were divided into three groups, one group receiving a cereal-legume (35 g/day) blended flour with 5 g Moringa leaf powder, the second group only receiving 5 g of *Moringa oleifera* leaf powder in form of “sprinkles” on top of the usual diet, and the third group receiving 35 g of cereal-legume blended complementary food without *Moringa oleifera* leaves powder. The analysis revealed no significant difference in hemoglobin status or growth parameters between the three groups. This finding might be explained by several factors, including the recruitment of healthy children without treatment of infection prior to intervention, thus leaving the possibility of sub-clinical infections affecting hemoglobin concentrations open, and anemia prevalence at baseline ranging from 53 to 64% in the three groups. The authors hypothesized that the amount of added Moringa (5 g) was too low and the period of intervention (4 months) might have been too short to see any improving effects in hemoglobin status in healthy children. The added amount of 5 g Moringa consists of ~1.1 mg of iron, which only represents 10% of the daily iron needs recommended by the WHO/FAO for the age group studied (24). Furthermore, no data collection on the use of other vitamin supplementations during the intervention period and a possible contribution of low iron bioavailability due to the presence of phytic acid in *Moringa oleifera* leaves represent additional limitations of the study. The latter was also discussed and observed in the study of Gallaher et al. (25), who evaluated the iron bioavailability of *Moringa oleifera* leaf powder applied to rats. Irrespective to these points, a broader investigation of other iron parameters, such as transferrin receptor or serum ferritin should have been favored instead of observing a single biomarker (hemoglobin). Additionally, a lower adherence in the two groups receiving *Moringa oleifera* leaves powder (61%) was shown compared to the third group who consumed no leaf powder (80%). The adherence was measured by weighing and documenting the amount of leftovers of the supplied study food. This outcome is in accordance with a recent report which compared the acceptability of different products, such as biscuits, cakes, soups or dairy products with a varying amount of bitter-tasting *Moringa oleifera* leaf powder (6, 26). The evaluation studies were conducted with adult study participants (26). In addition to the high chlorophyll content of the leaves which lead to an intense green color and metallic off taste, the strong herbal smell was discussed to lower the acceptability of Moringa fortified products.

A recent study conducted in iron depleted rats detected a very low bioavailability of iron in air-dried *Moringa oleifera* leaf powder (25). For analyzing iron bioavailability, the hemoglobin regeneration efficiency (HRE) ratio assay was used. The HRE ratios were 2.6 and 1.0 for adding ferrous sulfate and ferric orthophosphate to a purified diet, respectively after 9 days of intervention. An iron repletion for 13 days revealed values of 2.7 when ferrous sulfate was added and 1.2 when iron was added in form of the poorly-absorbed ferric orthophosphate. Consuming a Moringa-rich diet consisting of 38.7 g Moringa/kg diet only showed a HRE ratio of 0.05 and 0.4 after 9 and 13 days, respectively. The HRE ratio decreased from 0.59 after 9 days of repletion to 0.4 after 13 days of iron repletion when Moringa leaf powder was added to a traditional Ugandan recipe, mainly containing rice and ground nuts (5.4 g Moringa/kg diet). The authors assumed that the low bioavailability was caused by the high phytate content of nearly 64 mg/g dry weight existing in the dried leaves (25). In another 3 month intervention study, Senegalese lactating women showed no significant changes of plasma ferritin levels after dietary supplementation of 100 g *Moringa oleifera* leaf powder per week (27). The plasma ferritin levels of 13 women in the Moringa group (n=33) and 14 women in the control group (n=31) were below 12 μg/L at baseline and were classified as iron deficient. After 3 months of intervention, plasma ferritin levels did not change significantly in the Moringa group, whereas only two women showed iron deficiency in the control group who received two tablets weekly, each equivalent to 130 mg of elemental iron plus 0.5 mg folic acid. In this study, the presence of polyphenols in *Moringa oleifera* leaf powder and the subsequent formation of non-bioavailable polyphenol-iron complexes was suggested to explain low iron bioavailability (27).

### Influences of Processing on Vitamin Content

*Moringa oleifera* leaves are promoted for their high vitamin content. Compared to 100 g of oranges, which contain ~53 mg vitamin C, Saini et al. (21) showed an ascorbic acid content of 271 mg in fresh *Moringa oleifera* leaves (28). Including a realistic consumption quantity of 40 g, which was estimated as an average daily intake from the recommendations on commercially available products (29), would yield 108 mg vitamin C and ≈200 g orange pulp in this equation. The α-tocopherol content of 37 mg per 100 g found in fresh Moringa leaves was comparable to the amount of 41 mg found in 100 g sunflower oil as stated in the USDA Food Databank (28). Furthermore, Saini et al. (21) revealed a trans-lutein content and trans-β-carotene content of 36.9 mg/100 g and 18.3 mg/100 g, respectively, in fresh leaves. Hence, this carotenoid content is higher than the amount stated for raw carrots, which contain ~8 mg of β-carotene in 100 g (28). However, Saini et al. (21) investigated the retained amounts of vitamins in *Moringa oleifera* leaves when different drying methods are applied, since it is often consumed in a dried form. The highest preservation of these phytoconstituents was obtained by lyophilisation, with a true retention of 89.9% of trans-β-carotene, 51.3% of trans-lutein, 86.7% of α-tocopherol and 97.8% of ascorbic acid (analyzed in triplicates with a standard deviation <5%). With cabinet tray drying, a similar retention rate of trans-β-carotene was obtained, whereas micro-oven-drying, oven-drying and sun-drying decreased the preserved trans-β-carotene content significantly, with sun-drying having the highest impact. A considerable loss in carotenoids should be taken into account when consuming sun-dried Moringa leaves, which is the usual way of drying leaves in African households (21). When sun-dried, only 54.4 mg/100 g trans-β-carotene were retained compared to 82.1 mg/100 g after lyophilisation. The authors stated that α-tocopherol content was the least affected phyto-constituent by all the drying methods, followed by the total phenolic content. Ascorbic acid on the other hand, could only be preserved efficiently by lyophilisation (97.8%) and micro-oven-drying (64.4%). All other drying techniques reported significantly lower total retention of 43.6% by cabinet tray drying, 40.2% by oven-drying and 37.6% by sun-drying (21). To conclude, especially ascorbic acid and carotenoid contents suffer from sun-drying, which would clearly recommend an, albeit more elaborate, lyophilisation procedure. However, the apparent gaps in Table 3 warrant further, comprehensive investigations on the effects of processing on the vitamin content of all edible Moringa parts and their respective treatments.

### Anti-nutrients

Phytic acid was assumed to negatively affect the iron bioavailability in the study of Gallaher et al. (25), who detected 6.4 g/100 g dry weight of phytic acid in dried *Moringa oleifera* leaves. In contrast, Gidamis et al. (30) reported much lower amounts of phytic acid, with 0.23 mg/100 g found in cooked leaves. Additionally, the authors did not observe a significant difference in phytate levels in pods as well as in leaves after cooking. Phytic acid contents of raw and cooked *Moringa oleifera* pods were 0.25 mg/100 g and 0.24 mg/100 g, respectively. After cooking, a decrease of tannins could be shown for both, pods and for leaves (Table 4).

**Table 4:** Anti-nutrient contents in mg/100 g^1^.

**Sample**	**Treatment**	**Phytate**	**Tannins**	**Saponins**	**Oxalate**	**Trypsin inhibitor**	**References**
Leaves (flour)	Dried	–	2.1		1,050	1.45^2^	(16)
Seeds (flour)	Raw	3.3	9.8	0.1	2.9	na^3^	(17)
	Boiled	3.9–11.7	1.7–7.8	0.4–0.5	2.5–3.1		
	Roasted	3.9–5.6	1.2–7.4	0.2–0.4	2.9–3.6		
Leaves	Raw	0.3^a^	0.22^a^	–	na	nd^3^	(30)
	Cooked	0.2^a^	0.16^bc^			nd^3^	
Immature pods	Raw	0.2^a^	0.2^ab^	–	na	0.3^a^	(30)
	Cooked	0.2^a^	0.1^c^			0.3^a^	

1 *Values are presented as means or range, different superscript letters per column represent significant differences (p ≤ 0.05)*.

2 *in TUI/g*.

3 *na = not analyzed; nd = not detected*.

A phytochemical evaluation of *Moringa oleifera* seeds was conducted by Mbah et al. (17) in which the maximum contents of phytate, tannins and saponins reached 11.7 mg/100 g, 9.8 mg/100 g and 0.5 mg/g, respectively. The authors additionally evaluated oxalate contents between 2.5 and 3.6 mg/100 g. Even higher oxalate contents were found in dried *Moringa oleifera* leaves, reaching 1,050 mg/100 g (16). Additionally, trypsin inhibitor with a total of 1.45 TUI/g could be detected (16). The trypsin inhibitor content of 0.3 mg/100 g dry weight found in *Moringa oleifera* pods was not affected by cooking (30). As depicted in Table 4, the overall lower anti-nutrient contents favor leaves and pods over seeds for consumption. However, more complete data on different products and treatments as well as the comparison to their respective nutrient contents are still required.

### Contaminants

#### Heavy Metals

Regarding the contamination of Moringa products with heavy metals, a brief glance into the research fields of biosorption and phytoremediation might raise some concerns. In detail, besides frequently proposed uses of *Moringa oleifera* seeds in the biosorption of heavy metals, a few studies also highlighted the phytoremediation potential for contaminated soil, e.g., as described for cadmium (31) and lead (32). As such, the potential hyper-accumulation of heavy metals from the cultivation sites in edible Moringa parts should be considered, along with a thorough monitoring of the soil and Moringa products in the future.

Aissi et al. (29) evaluated the presence of lead and cadmium in *Moringa oleifera* leaf powders in 24 samples, available from 12 different national and international producers in Benin. The average lead content reached 1.53 mg/kg and a total of 58.3% of the products contained higher levels than the allowed maximum limits set by the European Commission Regulation (EU) No 1881/2006 of 0.3 mg/kg for leafy vegetables (33). Higher lead contents were reported by Limmatvapirat et al. (34) for products purchased in Thailand, with an average value of 2.45 mg/kg for all investigated products and 1.98 mg/kg for leaf powder. Additionally, the authors reported noteworthy levels of arsenic, cadmium and mercury, with 0.362 mg/kg, 0.122 mg/kg and 0.087 mg/kg, respectively. The overall highest contents for the assessed heavy metals were found in tea leaves. While the average cadmium value was below the maximum limit of 0.2 mg/kg defined in the EU regulation No 1881/2006 (33), especially leaf capsule samples exceeded this threshold with contents up to 0.6 mg/kg. Regarding arsenic and mercury, threshold values are available for rice and rice products for arsenic and complementary foods for mercury, with 0.10–0.30 and 0.10 mg/kg, respectively. In light of this, the reported arsenic levels of up to 1.57 mg/kg particularly warrant thorough monitoring.

In comparison, Aissi et al. (29) reported a higher mean cadmium content of 0.25 mg/kg. Although the difference of this value to the maximum allowed limit of 0.2 mg/kg was not statistically significant, the authors stated exceeding levels of cadmium in 75% of the samples, with values up to 0.35 mg/kg. Nevertheless, it was concluded that there is only a low risk of heavy metal intoxication for consumers in general, as the DJE (dose journalière d'exposition apportée par l'alimentation générale) for Moringa was estimated to account for <2% compared to the consumption of other food. The average daily amount of Moringa leaf powder consumed by adults and children was estimated with 40 g according to the recommendations on the packaging.

To summarize, the lead and cadmium contamination through Moringa leaf powders was not categorized as a risk for consumers in general (29). However, the promotion of using *Moringa oleifera* products for treating malnutrition or as a product supporting a healthy lifestyle should still be taken with caution: Individual samples exceeded the existing threshold values of several heavy metals considerably (29, 34) and, when coupled to an increasing daily consumption, a more significant contribution of Moringa products to the total daily intake of these contaminants could be conceivable in the future. Special care also needs to be taken regarding the intended use of Moringa preparations, as food for infants has significantly lower allowed concentrations, e.g., 20 μg/kg for lead and cadmium (33).

#### Polycyclic Aromatic Hydrocarbons

Polycyclic aromatic hydrocarbons (PAHs) are a class of ubiquitous and persistent organic compounds, some displaying genotoxic and/or carcinogenic activity. Alongside the 16 identified priority PAHs, the amount of Benzo(a)pyrene as well as the sum of PAH4 are often applied as indicators in risk assessment (35, 36). PAHs are formed in various natural and synthetic processes through pyrolysis during the incomplete combustion of organic substances, and may therefore contaminate food through numerous ways—from atmospheric deposition or contaminated soil to industrial food processing and cooking (37). Besides deposition and soil contamination, e.g., a previously described source for tea leaves from drying with combustion gases (38) could also contribute to PAH content of dried Moringa products.

A recent evaluation of PAHs in Moringa herbal tea revealed a ∑_16_PAH content of 5.03 ± 0.84 μg/kg, whereby 5-ring and 6-ring PAHs were the predominant forms (35). Compared to two or three ring aromatic systems, these molecules are more toxic and less susceptible to degradation processes. This study evaluated the PAH contents in 23 teas, including green, black and herbal teas, in Nigeria. The ∑_16_PAH content of herbal teas ranged between 4.71 and 79.6 μg/kg, whereas the content found in green and black teas were between 1.63–73.5 and 12.5–27.0 μg/kg, respectively (35). For ∑_16_PAH no maximum levels have been established so far in the EU. However, the revealed level of 5.03 ± 0.84 μg/kg in Moringa herbal tea is still lower than the maximum levels of 50 and 10 μ/kg set for ∑_4_PAH and benzo(a)pyrene in dried herbs, respectively (36). Nevertheless, due to the ubiquity of PAHs and various contamination sources, their thorough screening remains necessary.

#### Mycotoxins

Mycotoxin analysis in *Moringa oleifera* products are very limited, but the outcome of a recent study conducted by Aristil et al. (39), strengthens the need for a more profound mycotoxin evaluation on *Moringa oleifera* products, and in particular on seeds. The main goal of this study was to evaluate the contamination of toxigenic fungi in nine maize, three moringa and six peanut seed samples collected in Haiti. Furthermore, to determine *via* the ELISA technique if the *Aspergillus* section Flavi strains have the potential of producing aflatoxins AFB1 and AFG1. The analysis showed that 71% of the detected fungi in moringa seed samples belonged to *Aspergillus* spp., *Fusarium* spp. and *Penicillium* spp., which are potential mycotoxin producers, whereby other fungi made up 28% of the isolated mycobiota. In comparison, these three taxa made up 90% of the fungi in maize samples, and 51% of the mycobiota found in peanut seeds belonged to *Aspergillus* spp. An monoconidial isolation of *Aspergillus* spp., *Fusarium* spp. and *Penicillium* spp. was carried out in pure culture afterwards. Five isolates of *Aspergillus* section Flavi strains from Moringa seeds were tested for aflatoxin production, whereby three isolates were capable of producing AFB1, and the other two isolates either produced both mycotoxins AFB1 and AFG1 or no aflatoxin, respectively. However, the aflatoxin contamination in the analyzed Moringa samples ranged from non-detectable (LOD: 2.1 μg/kg) to a maximum of 700 μg/kg. Taking the maximum limit of 10 μg/kg for almonds and other kernels for direct human consumption as a reference, the aflatoxin contamination in Moringa samples can be alarmingly high (36).

### Safety Assessment

Although an increasing number of investigations addressing the positive health effects of *Moringa oleifera* are conducted, studies about safety and toxicity evaluations are largely lacking.

In the review of Stohs and Hartman (12), *Moringa oleifera* leaves consumed in its different preparations (extract, powder) are considered relatively safe as no adverse effects have been reported in association with human studies until now. Genotoxicity was only observed when rats were administered with an aqueous *Moringa oleifera* leaf extract at a supra-supplementation level of 3,000 mg/kg body weight (40). However, an amount of 1,000 mg/kg body weight, which is still higher than commonly consumed doses, did not exhibit genotoxic effects (40). Administration of a methanolic extract of *Moringa oleifera* leaves at a dose of 200 and 400 mg/kg body weight fed to rats over a period of 8 weeks indicated negative effects on hepatic and renal function (41). Nevertheless, this amount is very unlikely to be consumed on a regular basis, since 400 mg methanolic leaf extract/kg body weight would be equivalent to 12 g leaves/kg body weight (41). Furthermore, the amounts given and results achieved from rodent studies are not transferable one-to-one to humans and need to be extrapolated. An amount of 12 g leaves/kg body weight for a rat would mean ~156 g Moringa leaves for an 80-kg adult, which is higher than the commonly recommended doses of ~40 g/day (12, 29).

Several reports about the potential of *Moringa oleifera* leaves to influence fertility, contraception and the reproductive status are existing (42, 43). For example, Attah et al. (42) analyzed the *in vivo* and *in vitro* contraceptive and abortifacient potential of Moringa leaf powder when administered before and after mating of Wistar rats. Uterine contractility was reported for hot as well as for cold extracts of *Moringa oleifera* leaf extract, whereby the highest activity was shown for cold aqueous extracts. This might be caused by the presence of metabolites formed upon digestion and absorption which are degraded at higher temperatures. A dose of 58 mg/kg body weight of cold aqueous extract or 50 mg/kg body weight of a hot aqueous extract which was administered before mating, resulted in 100% infertility and 83.3% infertility, respectively. An amount of 58 mg/kg body weight was stated to be comparable to 250 mg dry plant product. When the Wistar rats received these doses after mating, an 80% abortion activity for cold, and 50% abortion for hot aqueous extracts was observed. No abortion could be shown in animals of the control group, which only received distilled water, whereas a significantly altered morphometry growth was shown by pups delivered by Wistar rats in the hot aqueous extract group.

A study conducted with rabbits investigated differences in reproductive hormone status between female and male rabbits when they received 0–15 g/kg *Moringa oleifera* powder over a period of 12 weeks (43). The follicle stimulating hormone (FSH) was highest in the control group (2.7 ± 0.3 IU/mL) compared to the three Moringa treatment groups which showed serum FSH levels of 1.1 ± 0.1, 0.9 ± 0.1, and 0.6 ± 0.1 IU/mL when the female rabbits were administered with 5, 10 or 15 g/kg *Moringa oleifera* powder, respectively. Contrary, FSH levels of rabbit bucks were higher after receiving 15 g/kg Moringa powder (1.9 ± 0.6 IU/mL), than in the control group (0.9 ± 0.3 IU/mL). Also, semen quality and sperm count improved with an increasing amount of dietary *Moringa oleifera* powder. With an additional investigation of other hormones, such as luteinizing hormone (LH), progesterone and testosterone, the authors could show a supporting effect of *Moringa oleifera* powder on the fertility of bucks, although it led to infertility in female rabbits (43).

When it comes to dietary supplementation with *Moringa oleifera*, the powdered form is rather consumed than the Moringa extracts. However, since no standardized method of aqueous and alcoholic extraction has been established, high variations in the extract constituents have to be expected which do not allow direct comparisons of scientific data from animal studies and their extrapolation to humans.

## Conclusion

Even though several attempts have already been made to evaluate the composition and safety of *Moringa oleifera* products, variations in processing, preparation and extraction methods impede not only the direct comparison of data, but also the formulation of guidelines for dietary intakes. Despite these challenges, the dietary intake of *Moringa oleifera* leaves has been considered as safe at relatively high doses, and should be re-evaluated in light of individual constituents, e.g., heavy metals and PAHs, as well as mycotoxins. High levels of anti-nutrients and contaminants indicate the need for a more profound analysis accompanied by contingent legal regulations for the dietary intake of *Moringa oleifera* products.

## Author Contributions

SG reviewed the literature and drafted the manuscript. PP reviewed the literature and c-drafted the manuscript. VS and KK reviewed the literature, conceptualized, and revised the manuscript. All authors approved the finalized version of the manuscript.

## Funding

The work presented has been funded by the University of Vienna, the Leibniz Institute for Food Systems Biology, and the Technical University of Munich.

## Conflict of Interest

The authors declare that the research was conducted in the absence of any commercial or financial relationships that could be construed as a potential conflict of interest.

## Publisher's Note

All claims expressed in this article are solely those of the authors and do not necessarily represent those of their affiliated organizations, or those of the publisher, the editors and the reviewers. Any product that may be evaluated in this article, or claim that may be made by its manufacturer, is not guaranteed or endorsed by the publisher.
